# Patients’ perspectives on deprescribing in swedish primary care: an exploratory survey study

**DOI:** 10.1080/02813432.2026.2636593

**Published:** 2026-03-01

**Authors:** Naldy Parodi López, Hans Thulesius, Stina Mannheimer, Katharina Tabea Jungo, Kristie Rebecca Weir, Zsofia Rozsnyai, Sven Streit, Renata Vidonscky Lüthold

**Affiliations:** ^a^ Unit of Clinical Pharmacology, Department of Pharmaceuticals, Sahlgrenska University Hospital, Gothenburg, Sweden; ^b^ Department of Pharmacology, Sahlgrenska Academy, University of Gothenburg, Gothenburg, Sweden; ^c^ Department of Medicine and Optometry, Linnaeus University, Kalmar, Sweden; ^d^ Institute of Health and Care Sciences, Sahlgrenska Academy, University of Gothenburg, Gothenburg, Sweden; ^e^ Institute of Primary Health Care (BIHAM), University of Bern, Bern, Switzerland; ^f^ Division of Pharmacoepidemiology and Pharmacoeconomics and Center for Healthcare Delivery Sciences (C4HDS), Department of Medicine, Brigham and Women′s Hospital and Harvard Medical School, Boston, Massachusetts, USA; ^g^ Sydney School of Public Health, Faculty of Medicine and Health, University of Sydney, Sydney, Australia

**Keywords:** Aged, deprescribing, older adults, polypharmacy, primary health care, survey

## Abstract

**Background:**

Understanding patients’ perspectives on their medications is important to facilitate collaborative deprescribing and shared decision-making.

**Aim:**

To explore older patients’ attitudes towards having their medications deprescribed and facilitators for deprescribing in Swedish primary care.

**Methods:**

Swedish primary care patients (≥ 65 years and taking ≥5 medications) responded to an anonymous survey on their attitudes towards deprescribing (June 2022-December 2023).

**Results:**

Out of 101 patients (45% women), 81% were satisfied with their medications (*n* = 82), 78% would be willing to stop or reduce ≥1 medication if their physician said it was possible (*n* = 79), and 27% (*n* = 27) wanted to deprescribe ≥1 medications from their medication lists (with 48 unique medications being mentioned). Side effects associated with the medication was the most commonly stated reason for wanting to deprescribe, mentioned by 52% (14/27 patients). Patients most often wanted to deprescribe blood glucose lowering medications, mentioned by 26% (7/27 patients). Most frequent patient-perceived facilitators for deprescribing were the support from their general practitioner (GP) (68%), a plan for deprescribing (38%), the possibility of restarting the medication if needed (23%), and having an alternative medication (22%).

**Conclusions:**

While most older adults with polypharmacy in Swedish primary care would be willing to deprescribe if suggested by their physician, many did not want to have any medication deprescribed. This difference highlights the need to clarify patients’ preferences when discussing medication changes. Support from the GP was identified as an important facilitator, demonstrating the importance of patient-GP communication and shared decision-making in deprescribing.

## Introduction

In Sweden, polypharmacy (commonly defined as ≥5 medications [1]) is present in almost half of patients aged ≥65 years, both among patients in general [2] and in primary care [3]. Polypharmacy increases the risk of adverse drug reactions, drug interactions and hospitalisations [4,5]. In 2004, the Swedish National Board of Health and Welfare launched indicators of prescribing quality in older adults to support identification of potentially inappropriate medications [6]. Some of these indicators are incorporated into a Swedish decision support system integrated in electronic medical records [7]. Once potentially inappropriate medications have been identified, either by relying on their own expertise and experience or by the use of decision support, physicians may consider deprescribing, which is the process of identifying and discontinuing or reducing medications that are no longer beneficial or might cause harm, considering the patient’s care goals, function, values and preferences [8].

As many community-dwelling older adults have polypharmacy [9], understanding this population’s views regarding deprescribing is essential to improving its implementation. Previous studies from Scandinavian countries, mostly conducted in Denmark, focused on different groups’ overall willingness to deprescribe. The groups included psychiatric patients [10], nursing home residents [11], patients with limited life expectancy [12], and patients from outpatient clinics [13]. Other studies focused on deprescribing specific medications, such as proton pump inhibitors or preventive medicines [14,15]. However, there is a lack of Swedish studies on the overall attitudes towards deprescribing of older community-dwelling patients attending primary care health centres.

A recent multi-country survey study conducted by the authors of this study found that 44% of older adults wanted to stop or reduce ≥1 medication from their own medication list, and medications for cardiovascular diseases were the most often mentioned by participants wanting to deprescribe [16]. Using a subsample from this multi-country survey study, we aimed to explore older patients’ attitudes towards having medications deprescribed in the Swedish primary care settings, focusing on which specific medications they wanted to have deprescribed and why, as well as on patient-perceived facilitators for deprescribing.

## Materials and methods

### Study design and setting

The multi-country cross-sectional study investigating older adults’ attitudes towards deprescribing of medications in primary care settings in 14 countries was conducted from May 2022 to December 2023 [16]. In the present sub-study, we only report data from the Swedish subsample, which were collected between June 2022 and December 2023. The GPs were instructed to recruit patients consecutively if they were aged ≥ 65 years and regularly (daily or on most days of the week) taking ≥5 medications (definition of polypharmacy [1]), and to invite them to complete the survey. The full details of the multi-country survey study have been published elsewhere [16,17].

### Study population

In the Swedish subsample, patients (aged ≥ 65 years and regularly taking ≥5 medications) attending GPs at four primary care centres (one urban, two suburban, and one suburban/rural) in Region Kalmar and Region Västra Götaland were included. These centres were staffed with a total of 41 physicians. Patients unable to give informed consent were not eligible to participate.

### Data collection and management

Each patient responded to an anonymous survey [16], which involved study-specific questions developed by the study team and questions from validated instruments [16,18–21]. The German version of the questionnaire was piloted with six patients in Switzerland. The English version of the questionnaire was translated and back-translated into Swedish and cross-culturally adapted. Patients could respond to the survey on paper or online, at home or on-site. Details of the survey can be found in the supplement section of this paper. In this study, we used data on patients’ sociodemographic and health characteristics, number of regular medications, medications that patients would like to have deprescribed and reasons for this, facilitators for deprescribing, one global question from the revised Patient Attitudes Towards Deprescribing (rPATD) questionnaire ‘If my doctor said it was possible I would be willing to stop one or more of my regular medications’ [18], as well as questions on trust in their physicians [19]. Patients who answered ‘yes’ to the question ‘Thinking about your current medication list, are there any medications that you would like to stop taking or reduce the dose of?’ were considered to *want* deprescribing. Patients who answered ‘strongly agree’ or ‘agree’ to the 5-point Likert scale question ‘If my doctor said it was possible I would be willing to stop one or more of my regular medications’ were considered *willing* to deprescribe. Patients also identified their reasons for wanting or not wanting deprescribing from a predetermined list. Facilitators of deprescribing were assessed by the question ‘What would help you to stop or reduce the dose of a medication?’, for which patients could select their responses based on a predetermined list that was informed by previous research [22]. Free-text responses involved names of medications (substances or brand names). These were categorized according to the Anatomical Therapeutic Chemical (ATC) classification, at the 5th and 3rd levels [23,24]. Combinations of substances were counted as one medication. Other free-text responses were written verbatim. Unclear responses were discussed within the research group to reach consensus.

### Statistical analysis

Descriptive results are presented as numbers (percentages) and/or median (interquartile range (IQR), or range). No sample size calculations were conducted, as the sample size for this study was predetermined as part of a larger study sample [17]. We compared patients wanting and not wanting deprescribing using the chi-square or Fisher test for categorical data and the Mann-Whitney U test for discrete data. The names of all medications that patients wanted to stop or reduce the dose of were described as counts and percentages. We used descriptive statistics to analyse factors that could facilitate deprescribing. A two-sided P-value of <0.05 was considered statistically significant. Statistical analyses were performed using SPSS Statistics for Windows, version 28.0.1.0 (IBM Corp., Armonk, NY, USA).

## Results

In total, 101 patients responded to the questionnaire, of which 45 (45%) were women. The median number of regular medications used was 6 (range 5–13, IQR 5–7). Ninety-five (95%) patients were treated with 5–9 regular medications, and six (6%) had ≥10 regular medications. Regarding patients’ characteristics, we did not find any difference between patients wanting and not wanting to deprescribe (Table 1).

**Table 1.** Socio-demographic and health characteristics of swedish primary care patients (*n* = 101; all ≥65 years of age and taking ≥5 medications) by their wish to deprescribe.

Characteristics^a^		Thinking about your current medication list, are there any medications that you would like to stop taking or reduce the dose of?		*p*-value^b^
		Yes (n = 27)	No (n = 74)	
Gender	Female	13 (48)	33 (45)	0.75
	Male	14 (52)	41 (55)	
Living area	Urban	20 (74)	54 (73)	0.91
	Suburban or rural	7 (26)	20 (27)	
Home setting	Living alone	10 (37)	28 (38)	0.94
	Living with other person(s)	17 (63)	46 (62)	
Type of residence	Owned	24 (89)	58 (78)	0.23
	Rented	3 (11)	16 (22)	
Education level	None or primary school	4 (15)	21 (28)	0.20
	Secondary school or higher education	23 (85)	53 (72)	
Confidence in filling out medical forms^c^	Yes	26 (96)	72 (99)	0.47
Balancing finances^d^	With difficulties	4 (15)	7 (9)	0.48
	Without difficulties	23 (85)	67 (91)	
Self-rated health^e^	Good health	11 (41)	38 (51)	0.35
Born in Sweden	Yes	24 (89)	69 (93)	0.44
Have a regular doctor	Yes	23 (85)	63 (85)	1.00
Medication preparation, self-prepare	Yes	26 (96)	70 (95)	1.00
Number of regular medications, median (range)		6 (5–13)	6 (5–12)	0.22

Values are presented as n (percentage) if not stated otherwise.

^a^ Missing *n* = 1, in ‘Confidence in filling out medical forms’.

^b^ According to chi-square test or Fisher-test for categorical data, and the Mann-Whitney test for discrete data.

^c^ Confidence in filling out medical forms was dichotomised considering ‘a little bit’, ‘somewhat’, ‘quite a bit’, and ‘extremely’ as ‘yes’, and ‘not at all’ as ‘no’.

^d^ Balancing finances was dichotomised considering ‘with great difficulty’ and ‘with some difficulty’ as ‘with difficulties’, and ‘quite easily’ and ‘without any problems’ as ‘without difficulties’.

^e^ Self-rated health was dichotomised considering ‘good’, ‘very good’ and ‘excellent’ as ‘good health’, and ‘average’ and ‘poor’ as ‘not good health’.

Overall, 79 (78%) patients expressed willingness to stop one or more regular medications if their doctor said it was possible, and 82 (81%) were satisfied with their current medications.

When asked about their specific medication list, 27 (27%) patients wanted to have at least one medication deprescribed. These 27 patients reported 48 medications that they would like to have deprescribed, of which blood glucose lowering medications were mentioned most often by 7/27 (26%) patients (Table 2). The most common reason for patients wanting to have at least one medication deprescribed was suspected side effects, reported by 14 (52%) out of 27 patients (Table 2).

**Table 2.** Medications that swedish primary care patients ≥65 years of age would like to deprescribe (*n* = 27) and underlying reasons (*n* = 48).

ATC classification^a^	Medication^c^	Reasons^e^^,f^
Blood glucose lowering drugs, excluding insulins	Metformin (*n* = 4)	It causes side effects (*n* = 2); “Easy to forget the evening dose” (*n* = 1); “Normal glucose levels” (*n* = 1)
	Dapagliflozin (*n* = 1)	It causes side effects (*n* = 1); I do not like the medication (*n* = 1); The medication is too expensive (*n* = 1)
	Empagliflozin (*n* = 1)	NR
	Semaglutide (*n* = 1)	“Weight loss” (*n* = 1)
Lipid modifying agents	Rosuvastatin (*n* = 3)	It causes side effects (*n* = 2); The medication is too expensive (*n* = 1); I do not like the medication (*n* = 1)
	Atorvastatin (*n* = 2)	It causes side effects (*n* = 2)
Antithrombotic agents	Warfarin (*n* = 1)	“The thrombus in the heart is gone” (*n* = 1)
	Tinzaparin (*n* = 1)	“The treatment period will end” (*n* = 1)
	Clopidogrel (*n* = 1)	It causes side effects (*n* = 1)
	Apixaban (*n* = 1)	NR
Corticosteroids for systemic use, plain	Glucocorticoids^d^ (*n* = 2)	It causes side effects (*n* = 2)
	Prednisolone (*n* = 2)	It causes side effects (*n* = 1); I do not like the medication (*n* = 1)
Opioids	Morphine (*n* = 1)	“The need will end” (*n* = 1)
	Oxycodone (*n* = 1)	“Previous operation” (*n* = 1)
	Buprenorphine (*n* = 1)	“The need will end” (*n* = 1)
Drugs for peptic ulcer and GORD	Omeprazole (*n* = 2)	It causes side effects (*n* = 1); “Can I manage without it?” (*n* = 1)
Drugs for constipation	Lactulose (*n* = 1)	“The need will disappear” (*n* = 1)
	Macrogol, combinations (*n* = 1)	“The need will disappear” (*n* = 1)
Beta blocking agents	Bisoprolol (*n* = 2)	It causes side effects (*n* = 1); “I become so slow” (*n* = 1)
Selective calcium channel blockers with mainly vascular effects	Amlodipine (*n* = 1)	It causes side effects (*n* = 1)
	Felodipine (*n* = 1)	It is inconvenient for me to take the medication (*n* = 1)
Other analgesics and antipyretics	Paracetamol (*n* = 2)	It is inconvenient for me to take the medication (*n* = 1); “The pain will cease” (*n* = 1)
Antidepressants	Sertraline (*n* = 1)	It causes side effects (*n* = 1)
	Mirtazapine (*n* = 1)	NR
Antiarrhythmics, class I and III	Dronedarone (*n* = 1)	I do not like the medication (*n* = 1)
Antiadrenergic agents, peripherally acting	Doxazosin (*n* = 1)	It causes side effects (*n* = 1)
ACE inhibitors, plain	Ramipril (*n* = 1)	“Risk for kidney injury” (*n* = 1)
ARBs, plain	Candesartan (*n* = 1)	It is inconvenient for me to take the medication (*n* = 1)
Antiinflammatory and antirheumatic products, non-steroids^b^	Diclofenac (*n* = 1)	“I want to manage without it” (*n* = 1)
Urologicals	Mirabegron (*n* = 1)	It is inconvenient for me to take the medication (*n* = 1)
Drugs used in benign prostatic hypertrophy	Finasteride (*n* = 1)	“Unknown function” (*n* = 1)
Hormone antagonists and related agents	Exemestane (*n* = 1)	It causes side effects (*n* = 1)
Antigout preparations	Allopurinol (*n* = 1)	NR
Drugs affecting bone structure and mineralisation	Alendronic acid (*n* = 1)	I do not like the medication (*n* = 1); It is inconvenient for me to take the medication (*n* = 1)
Dopaminergic agents	Levodopa/benserazide (*n* = 1)	It causes side effects (*n* = 1)
Hypnotics and sedatives	Zopiclone (*n* = 1)	“Addictive” (*n* = 1)
Adrenergics, inhalants	Beclometasone/formoterol/glycopyrronium (*n* = 1)	The medication is too expensive (*n* = 1)

ACE = angiotensin-converting enzyme inhibitors, ARB = angiotensin II receptor blockers, ATC = anatomical therapeutic chemical, GORD = gastro-oesophageal reflux disease, NR = not reported.

^a^ Reported according to the ATC 3rd level.

^b^ Unclear administration route.

^c^ Each patient could report up to four medications that they would like to reduce or to discontinue.

^d^ Specific name of glucocorticoids was not provided.

^e^ One drug could have multiple underlying reasons.

^f^ According to a multiple-choice question: ‘it causes side effects’, ‘I do not benefit from it’, ‘I do not like the medication’, ‘the medication is too expensive’, ‘it is inconvenient for me to take this medication’, ‘the tasks involved in taking the medication are stressful for me’, ‘I often forget to take this medication’, ‘other reason’. Directly cited free-text answers are enclosed in quotation.

Among the 74 patients who did not want to have any medication deprescribed, the most frequent reason for not wanting deprescribing was the perceived benefits of the medication, reported by 49 (66%) patients (Table S1). If their physician said it was possible, 55/74 (74%) were willing to deprescribe, 15/74 (20%) were unsure, and 4/74 (5%) were not willing to deprescribe.

When participants identified whom they would talk to about deprescribing, the most commonly selected options were their GP (*n* = 92; 91%); another specialist physician (*n* = 31; 30%); a pharmacist (*n* = 6; 6%), and family or friends (*n* = 2; 2%). When comparing those wanting versus not wanting deprescribing, contact with another specialist physician was more common among those wanting deprescribing (13/27 (48%) versus 18/74 (24%), *p* = 0.022). Only two patients, both among those not wanting deprescribing, reported that they would refer to their family or friends for such discussions. Regarding the patient-GP relationship, patients often reported high trust in their GP (82/101; 81%) (Figure S1) and feeling comfortable discussing medication changes with them (91/101; 90%).

When participants were asked what would help them to stop or reduce the dose of a medication, the most commonly selected options were the support of their GP (*n* = 69; 69%); a plan or instructions for stopping or reducing the dosage (*n* = 38; 38%); the option to restart the medicine if necessary or if the symptoms return (*n* = 23; 23%); an alternative medication (*n* = 22; 22%); alternatives such as lifestyle change or physiotherapy (*n* = 11; 11%); or other (*n* = 4; 1%).

## Discussion

While most Swedish older patients with polypharmacy would be willing to have their medications deprescribed if suggested by their physician, only a minority explicitly wanted to have one or more of their medications deprescribed. Blood glucose lowering medications were the most commonly mentioned for deprescribing. Among those who wanted to stop or reduce the dose of their medications, side effects associated with medications were reported by half of the patients as an underlying reason. Furthermore, the majority of patients identified their GP as an important facilitator in deprescribing.

The finding that 78% of older adults were willing to deprescribe if their doctor said it was possible is in line with three Danish studies [10,11,13] reporting high willingness to deprescribe among older patients (85-92%), as well as with two international systematic reviews [25,26]. This consistent evidence suggests that older patients are receptive to deprescribing when it is recommended by their GP, highlighting the GP’s central role in supporting patients in deprescribing decisions. However, system-level barriers, such as limited consultation time, fragmented care, and single-disease guidelines, may limit the feasibility of deprescribing strategies that involve patient-GP communication. In this context, shared decision-making is crucial to building patient trust and ensuring that deprescribing decisions reflect patients’ individualised preferences and health goals.

Regarding patients’ desire to deprescribe specific medications, 73% of the patients did not want to have any medication deprescribed from their medication list. This is in contrast to the result from the multi-country survey, where a lower proportion (56%) of older patients did not want to have one or more medications deprescribed [16]. This difference may, at least in part, be explained by the decrease in potentially inappropriate medications according to Swedish indicators [27], as well as high rates of appropriate medication according to physicians’ assessment [3]. Patients with appropriate medication plans may be less likely to want to change their medications. Nevertheless, the main reason for patients in our study not wanting deprescribing was medication benefits, which aligns with the multi-country survey [16] and with previous research that reported favourable perceptions of medications as one of the main barriers to deprescribing [28–30].

Different findings regarding patients *willing* to deprescribe if their doctor said it was possible and patients who *wanted* to have medications deprescribed suggest that different questions to assess deprescribing attitudes may capture distinct aspects of patient perspectives [16,31]. Therefore, caution is warranted when comparing deprescribing attitudes across studies. Moreover, not wanting to deprescribe medications should not be interpreted as the patients being opposed to deprescribing. Indeed, the majority of patients not wanting to deprescribe specific medications expressed their willingness to deprescribe if their doctor said it was possible. It is possible that patients are open to deprescribing if suggested by a doctor, but they do not have the instinct desire to change their medications. On the other hand, this contrast may suggest that willingness alone may not translate into action and highlights the importance of GP communication and support in turning willingness into meaningful deprescribing decisions. The distinction between wish and willingness may be relevant for comparisons between studies and reinforces the role of the GPs in guiding patients through deprescribing decisions.

The central role of GPs in leading deprescribing decisions is also reflected in our finding that support of GPs was identified as the most important facilitator in deprescribing. In addition, GPs were the healthcare professionals whom patients would contact most often when they wanted to discuss deprescribing. As physicians are the only profession with a full prescribing licence in Sweden [32], and considering that primary care plays a central role in the Swedish healthcare system, these findings may not be surprising. In Sweden, GPs are responsible for the follow-up of most patients, including those with complex conditions. Consistent with these findings, a previous systematic review has emphasised the critical role of communication between primary care practitioners and patients as a key enabler for deprescribing in primary care settings [33]. Swedish national efforts to improve prescribing practices since 2004 may have supported the GPs’ role in managing polypharmacy, and the integration of decision support systems offers a valuable opportunity to strengthen shared decision-making between GPs and patients in deprescribing.

In the multi-country survey, which included data from the present study [16], diuretics were the medications patients most commonly wanted to deprescribe, and diabetes medications were ranked fourth. In contrast, our study showed that, in Sweden, diabetes medications were the most frequently reported for deprescribing, even though the frequencies were low. This is in line with a study in the United States, in which about half of the older adults on glucose-lowering medication were willing to have their medication for diabetes deprescribed [34]. Side effects were the most frequently selected reason for patients wanting deprescribing. This is in line with previous studies, including the main publication of the multi-country survey [16], in which 46% of all patients mentioned side effects as the main reason for wanting deprescribing. In addition, a previous vignette study among older patients in the United States identified risk of side effects as the most common reason for deprescribing [35]. However, although patients in our study stated ‘side effects’, it is not certain if their symptoms were in fact side effects associated with the medications. Indeed, a temporal relationship between the medication intake and the adverse event does not always imply causality as there may be other reasons behind it. Yet, the fact that every fourth patient in this study wanted to have a medication deprescribed and half of these patients stated side effects as a reason for wanting deprescribing informs us about the need for partnership between patient and prescriber and shared decision-making on both prescribing and deprescribing [36]. Nevertheless, it is unclear if patients would have made different decisions if they had received more information about the benefits and risks of their medications. Given these uncertainties, the role of healthcare professionals – particularly GPs – becomes especially important in guiding and contextualising deprescribing discussions, supporting and educating patients to make informed decisions.

## Strengths and limitations

To the best of our knowledge, this is the first study focusing on patients’ attitudes towards deprescribing specific medications of a Scandinavian cohort of older primary care patients attending primary care medical centres. Including data from four primary health care centres (rural, urban, and suburban) staffed by more than 40 physicians improved the generalizability of our results for primary care. In addition, the consecutive recruitment of patients supports the representativeness of typical older primary care patients with polypharmacy in Sweden, and minimized selection bias. However, selection bias cannot be completely ruled out. For example, patients who did not present for a routine visit during the study period or those who were severely ill may have been missed, potentially leading to an under-representation of these groups. The non-randomised sample, and the overall high health literacy, good self-reported health, and low number of immigrants limit the overall generalisability of our findings and their representativeness for populations such as those with immigrant status and those with cognitive impairment. Due to feasibility reasons, we only collected information on whether patients were ≥65 years and did not assess their exact age, being unable to assess age distribution in our sample. Given the anonymous data collection via GPs in different practices, we were unable to track response rates. Another limitation is that we did not have the patients’ diagnoses and complete medication lists, therefore, we cannot verify whether the medications most mentioned for deprescribing corresponded to the medications mostly taken by the participants. Asking patients to self-report medications may have led to recall bias, as patients may have forgotten some medications. Patients may also have incorrectly attributed pre-existing symptoms to medication-related side effects.

## Implications

In Europe, the proportion of people aged 65 and older is projected to increase from 21% in 2022 to 33% in 2100 [37], reflecting longer lifespans. Therefore, the number of medications per patient can also be expected to increase, posing additional challenges for medication management in primary care. Our study contributes new knowledge regarding older patients′ views on deprescribing and adds to the scarce literature on this topic in Sweden. Our results can inform future research analysing why certain medications or medication groups are preferred for deprescribing, as well as future interventional studies focusing on specific medications for deprescribing. In addition, our results shed light on facilitators for stopping or reducing medications, which may support the recently explored need to develop and implement a Swedish decision support system for deprescribing [38]. In such efforts, it is crucial to understand the needs and preferences of individual parties involved. Furthermore, the distinction between patient wishing and willing to have medications deprescribed, and the GP’s role in guiding them in the process, warrants further exploration in future research.

## Conclusions

While most older patients with polypharmacy in Swedish primary care were willing to have their medications deprescribed if suggested by their physician, many did not want to have any medications deprescribed from their own medication list. Concerns about side effects were a common reason for wanting to stop or reduce a medication, and GP support was identified as an important facilitator in deprescribing. Our findings highlight the importance of clarifying patients’ preferences and involving them in discussions about medication changes. By involving patients and providing clear information about the potential benefits and harms of medications, GPs can support shared decision-making in deprescribing and promote patient-centred care.

## Supplementary Material

Parodi Lopez et al_supplementary material.docx

## Data Availability

The data for this study are available to other researchers on request. The data will be made available for scientific research purposes after the proposed analysis plan has been approved by the core study team [16,17]. Data and documentation will be made available through a secure file-exchange platform after approval of the proposal. In addition, a data transfer agreement must be signed (which defines obligations that the data requester must adhere to regarding privacy and data handling). For data access, please contact the corresponding author.
