# Supplementary material for: Patients’ perspectives on deprescribing in swedish primary care: an exploratory survey study
Source: Scand J Prim Health Care. 2026 Mar 1;44(1):2636593. doi: 10.1080/02813432.2026.2636593 (PMC12954800; doi:10.1080/02813432.2026.2636593)
Supplement: Parodi Lopez et al_supplementary material.docx [file IPRI_A_2636593_SM2052.docx]

**Supplementary material**

Supplement to: Parodi López N, Thulesius H, Mannheimer S, Jungo KT, Weir KR, Rozsnyai Z, Streit S, Vidonscky Lüthold R. Patients’ perspectives on deprescribing in Swedish primary care: an exploratory survey study. Scand J Prim Health Care. 2026.

**Table of content**

| Table S1 | Reasons for not wanting to stop or reduce the dose of any medication among Swedish primary care patients ≥ 65 years of age (n=74) | Page 2 |
| --- | --- | --- |
| Figure S1 | Distribution of level of agreement to statements about trust in one’s general practitioner (GP)^a^ among Swedish older (≥65 years) primary care patients (n=101) | Page 3 |
|  | Study questionnaire | Page 4 |

**Table S1** Reasons for not wanting to stop or reduce the dose of any medication among Swedish primary care patients ≥ 65 years of age (n=74)

| **Reason^a^** | **n (%)** |
| --- | --- |
| Beneficial medicine | 49 (66) |
| Doctors only prescribe medication(s) that are necessary | 35 (47) |
| Taking the medicine for a long time so it is better not to change | 27 (36) |
| Taking several medications every day is manageable | 16 (22) |
| The medication does not cause side effects | 11 (15) |
| Medication(s) are not expensive | 5 (7) |
| It is easy to take medications than to make healthy lifestyle changes | 1 (1) |
| Other reasons | 3 (4) |

^a^ Patients could give several reasons.

**Figure S1** Distribution of level of agreement to statements about trust in one’s general practitioner (GP)^a^ among Swedish older (≥65 years) primary care patients (n=101)


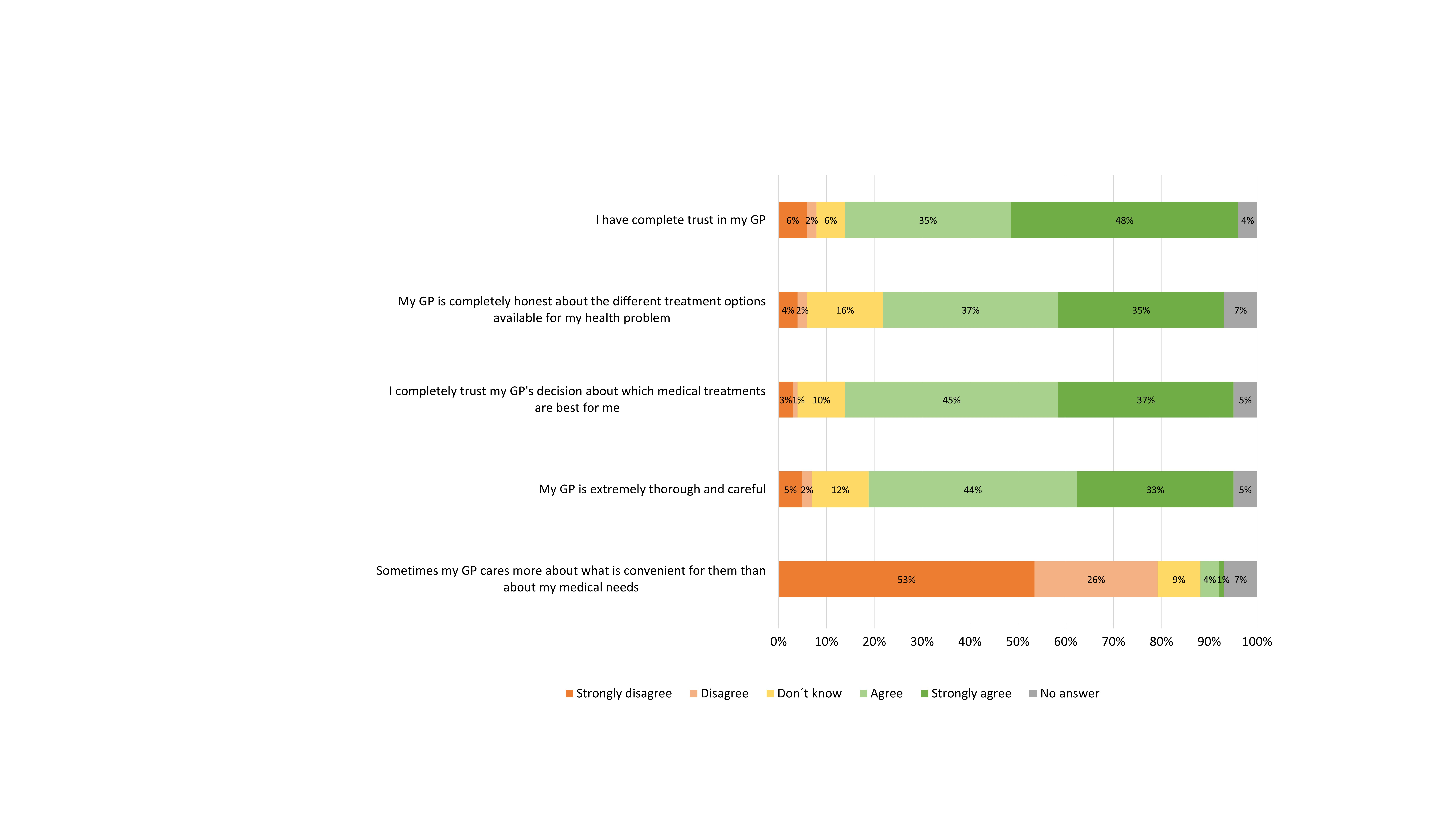


^a^ Trust questions from the Abbreviate Trust in the physician scale [19].

Missing: “I have complete trust in my GP”, n=5; “My GP is completely honest…for my health problem”, n= 7; “My GP is extremely through and careful”, n=5; “Sometimes my GP cares more about…than my medical needs”, n=7.

**Supporting Material 1. Study questionnaire**

**
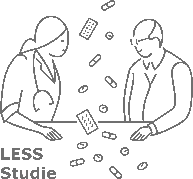
**

Dear Madam or Sir,

Thank you for your interest in participating in this study. It is being conducted by the Institute of Primary Health Care (BIHAM) of the University of Bern with collaborators from various European countries.

You were informed about this study in your GP’s practice and received this survey because your GP thinks you fulfill the inclusion criteria. We are conducting this study with adults who are 65 years or older and regularly take 5 or more medications. The aim of this project is to find out your opinion about stopping or reducing the dose of your medication. This is a survey only and no changes will be made to your medication as part of this project. The study ends after you have completed the survey.

In total, about 1000 patients from 14 countries are taking part in this survey. By taking part in this study, you contribute to better understanding how this group (adults aged 65 years or older, taking five or more medicines) think and feel about their medication. The results, which are based on the views of all the patients surveyed in this study, may be important in the future to help GPs care for their patients, by improving the process of stopping or reducing unnecessary medication.

Your answers will be kept anonymous. This means that neither your GP nor the study team can identify you or your answers.

You have the option of completing the questionnaire online or on paper. If you fill in the paper questionnaire, please return it to your GP in a sealed envelope. If you choose to complete the questionnaire online, please use this link or the QR-code above: ______

In this case, you do not need to return the hard copy of the questionnaire to your GP. We ask that you do not mention any names or addresses on the questionnaire to keep your anonymity.

This study is approved by the Ethics Committee of the Canton of Bern and complies with the legal requirements for research with anonymous medical data. The data will be securely stored electronically.

By answering "yes" to the question below and completing this questionnaire, you agree to participate in the study and the research team will collect your responses for the purpose of this study.

It will take about 15-30 minutes to complete the questionnaire.

Your help means a lot to us. Thank you again for taking the time to complete this questionnaire. If you have any questions or comments, please contact the study team:

Email: (Add email national coordinator)

Yours sincerely,

(Add name of national coordinator)

Prof. Sven Streit, PhD MD MSc

Principal Investigator of the LESS study

Institute of Primary Health Care (BIHAM)

University of Bern

**Informed consent**

Do you agree to participate in this study in which we will collect information about your medication use?

If you check "Yes", you agree to participate.

o Yes *(please continue to the next question)*

o No *(end of study participation)*

**1) Questions about the inclusion criteria**

1. How old are you (in years)?

- 65 years old or older *(please continue to the next question)*
- 64 years old or younger *(end of study participation, you are not eligible for this study*)

1. Do you regularly take 5 or more medications? (Regularly means: every day or most days for 30 days or more)

- Yes *(please continue to the next question)*
- No *(end of study participation, you are not eligible for this study*)

1. Do you live in (add country)

- Yes *(please continue to the next question)*
- No *(end of study participation, you are not eligible for this study*)

**2) Socio-demographic questions**

We will now ask you some questions to understand a bit more about you.

1. What is your gender?

- man
- woman
- other

1. What area do you live in?

- urban
- suburban
- rural

1. Do you live alone in your household?

- yes
- no

1. What is your living situation?

- Own your house or apartment
- Rented house or apartment

1. What is your highest completed education?

- None
- Primary school
- Secondary school (high school or vocational training)
- Third level education (university or equivalent training)

1. How do you make ends meet financially?

- With great difficulty
- With some difficulty
- Quite easily
- Without any problems

1. Where were you born?

- In the country where I currently live
- Other country: Please specify country___________________

1. What is your first language?

- Official language of the country where I live in
- Other language: Please specify language_________________

1. How confident are you filling out medical forms by yourself?

- Not at all
- A little bit
- Somewhat
- Quite a bit
- Extremely

1. In general, how would you describe your health today?

- Excellent
- Very good
- Good
- Average
- Poor

**3) Questions about your GP**

We will now ask you some questions about your GP.

1. Do you have your own GP/family doctor (definition: when you have a health problem, you usually consult the same family doctor, except in emergencies)?

- Yes
- No *(please go to Section 4) “questions about your use of medication”)*
- Unclear:
  - Reason: _______________________________________________

1. How long have you been seeing this GP?

- 0–9 years
- 10–19 years
- 20–29 years
- 30+ years

1. My GP is:

- man
- woman
- other

1. My GP’s practice is:

- In an urban area
- In a suburban area
- In the countryside

**4) Questions about your medication use**

Now we would like to learn more about your experiences with taking medications.

1. Do you prepare your medication by yourself?
   - Yes, I prepare and take it myself according to the prescription.
   - No, I receive support in preparing/taking my medication from relatives, home carers, or at the pharmacy for example.
2. Overall, I am satisfied with my current medications.

- Strongly agree
- Agree
- Don’t know
- Disagree
- Strongly disagree

1. How many different kinds of medications do you take regularly? (Regularly means daily or on most days of the week.) Please indicate the number of different kinds of medications.

*Number of different medications:* ___________________________________

**5) Questions about your attitude towards and decisions about medication**

Now we are going to ask you questions on your thoughts about stopping or reducing the dose of medicines.

1. If my doctor said it was possible I would be willing to stop one or more of my regular medications.

- Strongly agree
- Agree
- Don’t know
- Disagree
- Strongly disagree

1. I would like to try stopping one of my medications to see how I feel without it.

- Strongly agree
- Agree
- Don’t know
- Disagree
- Strongly disagree

1. Thinking about your current medication list, are there any medications that you would like to stop taking or reduce the dose of?

- Yes *(please continue to the next question)*
- No, I am not considering stopping or reducing the dose of any medication. *(Please go to Question 25)*

1. In the following table, please state the name(s) of the medication(s) that you would consider stopping or reducing, and the reason why.

*Any lines that are not applicable can be left empty.*

| **Name(s) of the medication(s) that you would consider stopping or reducing** | **Why did you choose this/these medication(s) to stop or reduce?**  *Please check all answers that apply* |
| --- | --- |
| **Name of the medication:**  __________________________ | - It causes side effects. - I do not benefit from it. - I do not like the medication. - The medication is too expensive. - It is inconvenient for me to take this medication. - The tasks involved in taking the medication(s) (e.g. blood glucose monitoring) are stressful for me. - I often forget to take this medication. - Other reason:___________________________ |
| **Name of the medication:**  __________________________ | - It causes side effects. - I do not benefit from it. - I do not like the medication. - The medication is too expensive. - It is inconvenient for me to take this medication. - The tasks involved in taking the medication(s) (e.g. blood glucose monitoring) are stressful for me. - I often forget to take this medication. - Other reason:___________________________ |
| **Name of the medication:**  __________________________ | - It causes side effects. - I do not benefit from it. - I do not like the medication. - The medication is too expensive. - It is inconvenient for me to take this medication. - The tasks involved in taking the medication(s) (e.g. blood glucose monitoring) are stressful for me. - I often forget to take this medication. - Other reason:___________________________ |
| **Name of the medication:**  __________________________ | - It causes side effects. - I do not benefit from it. - I do not like the medication. - The medication is too expensive. - It is inconvenient for me to take this medication. - The tasks involved in taking the medication(s) (e.g. blood glucose monitoring) are stressful for me. - I often forget to take this medication. - Other reason:___________________________ |

*After the table please continue to section 6 “additional questions about stopping medications and your willingness to do so”.*

1. You may not want to stop taking a medication or reduce the dose. Here are some reasons why. Which one(s) do you think are the most important reasons for not stopping a medication? (Please select all that apply)

- The medicine is beneficial.
- Taking the medicine for a long time so it is better not change it.
- Taking several medications every day is manageable.
- The medication does not cause side effects.
- Medication(s) are not expensive.
- Doctors only prescribe medication(s) that are necessary.
- It is easier to take medications than to make healthy lifestyle changes.
- Other reasons: _____________________________________________

**6) Stopping medications and your doctor’s involvement:**

We will now ask you some questions about how you would stop or reduce the dose of your medications with your doctor.

1. I feel comfortable talking to my doctor about changes to my medication

- Strongly agree
- Agree
- Don’t know
- Disagree
- Strongly disagree

1. Who would you talk to about stopping or reducing the dose of a medication*? (Please check all that apply)*

- GP
- Specialist
- Pharmacist
- Family and friends
- Other

1. What would help you to stop or reduce the dose of a medication? *(Please check all that apply)*

- A plan or instructions for stopping or reducing the dosage
- The support of my GP
- An alternative medication instead
- An alternative such as a lifestyle change, physiotherapy
- The option to restart the medicine if I feel I need to, or my symptoms return
- Other:______________________________________________

For each of the following, please select the statement that best aligns with your views.

1. What do you think about the medications you take?

- My medications are important, they keep me alive and help me live well.
- My medications do what they are supposed to do.
- I don’t really care much about my medications, I take them as my doctor tells me to.

1. How do you get information about your medications?

- My doctor and I talk about my medications together.
- I know about my medications – I ask my doctor or read the information leaflet or search online.
- I don't know much about my medications.

1. How do you make decisions about your medications?

- I want to be informed, but I trust my doctor to make decisions about my medications.
- I make decisions about the medications I take, or share the decision with my doctor.
- Other people (e.g. my doctor or my partner) make decisions for me about my medications.

1. What do you think about the idea of stopping or reducing the dose of one or more of your medications?

- I would not like to stop any of my medications or reduce the dose.
- I wish I did not take so many medications and I would stop or reduce the dose of my medications if I could.
- If my doctor said that it is possible to stop or reduce the dose of a medication that would be ok with me.

**7) Questions about your relationship to your family doctor**

33. This section is about your relationship with your GP and your trust in them. Please indicate how strongly you agree with each of the statements. There are no right or wrong answers.

|  | Completely disagree | Disagree | Don’t know | Agree | Completely agree |
| --- | --- | --- | --- | --- | --- |
| Sometimes my GP cares more about what is convenient for them than about my medical needs. |  |  |  |  |  |
| My GP is extremely thorough and careful. |  |  |  |  |  |
| I completely trust my GP's decision about which medical treatments are best for me. |  |  |  |  |  |
| My GP is completely honest about the different treatment options available for my health problem. |  |  |  |  |  |
| All in all, I have complete trust in my GP. |  |  |  |  |  |

**8) Final questions**

34. Did anyone help you with completing this questionnaire?

- Yes
  - If yes: Who? *(please check the answer that applies)*
    - Relatives
    - Friends
    - GP
    - GP practice staff
    - Other: _____________________________________________________
  - No

You had the opportunity to complete the questionnaire online or on paper. Please confirm that you **only completed one** of the versions of the questionnaire.

- “I confirm that I only completed one of the versions of the questionnaire.”

**Thank you for taking the time to complete the questionnaire.**

[Please return this questionnaire to your GPs office as soon as possible.]

If you have any questions, please do not hesitate to contact us.

Yours sincerely,

(Add national coordinator)

Prof. Sven Streit and the LESS Study team

*From: Vidonscky Lüthold R, Jungo KT, Weir KR, Adler L, Asenova R, Ares-Blanco S, et al. Older Adults' Attitudes Toward Deprescribing in 14 Countries. JAMA Netw Open. 2025;8(2):e2457498.*
